# Maxillomandibular Advancement Guided by the Barcelona Line, a Facial Soft-Tissue Reference, in Patients with Obstructive Sleep Apnea: A Feasibility Study

**DOI:** 10.3390/cmtr19040040

**Published:** 2026-10-09

**Authors:** Federico Hernández-Alfaro, Maria Cuevas-Nunez, Francesc Duran-Valles, Irene Méndez-Manjón, Maria Giralt-Hernando, Adaia Valls-Ontañón

**Affiliations:** 1 Department of Oral and Maxillofacial Surgery, Universitat Internacional de Catalunya (UIC), 08195 Barcelona, Spain; h.alfaro@uic.es (F.H.-A.); franky@uic.es (F.D.-V.); manjonirene@gmail.com (I.M.-M.); mgiralt@uic.es (M.G.-H.); avalls@uic.es (A.V.-O.); 2 Institute of Maxillofacial Surgery, Teknon Medical Center, 08022 Barcelona, Spain

**Keywords:** obstructive sleep apnea, Barcelona line, maxillomandibular advancement, orthognathic surgery

## Abstract

Maxillomandibular advancement (MMA) represents an effective surgical treatment for individuals with obstructive sleep apnea (OSA) when conservative therapies fail. However, variability in surgical planning methods limits predictability and standardization of outcomes. This retrospective cohort study evaluated clinical and polysomnographic outcomes of MMA planned using the Barcelona Line (BL), and to explore the relationship between BL-guided skeletal positioning and treatment response in adult patients with moderate to severe OSA. Twenty-six consecutive patients (N = 26) underwent standardized virtual planning, surgery, and follow-up, with preoperative and postoperative polysomnography and three-dimensional CBCT analysis. Skeletal movements, upper airway changes, and sleep-related outcomes were assessed. Mean advancement at pogonion was 19.0 ± 4.7 mm. The apnea–hypopnea index decreased significantly from 45.8 ± 11.6 to 6.6 ± 6.6 events/h (mean relative reduction of 85.6 ± 10.9%). Treatment success was achieved in 25/26 patients (96.2%; 95% CI 81.1–99.3) and cure in 22/26 patients (84.6%; 95% CI 66.5–93.9). Daytime sleepiness improved, with ESS scores decreasing from 12.2 ± 1.2 to 1.5 ± 0.4. Total upper airway volume increased from 26.46 ± 2.88 mm^3^ to 45.32 ± 5.41 mm^3^. BL-guided MMA is a feasible planning approach associated with substantial clinical improvement in selected OSA patients. The results likely reflect a combination of surgical advancement magnitude and patient selection. Comparative studies are required to determine the independent contribution of the BL.

## 1. Introduction

Obstructive sleep apnea (OSA) is a common disorder characterized by recurrent upper airway collapse during sleep, resulting in significant morbidity and increased perioperative risk, particularly in surgical populations [1]. Maxillomandibular advancement (MMA) is an established surgical treatment option for individuals with moderate-to-severe OSA who are intolerant of, or inadequately treated with, non-surgical therapies. Studies showed evidence supporting its efficacy and reducing the apnea–hypopnea index (AHI) [2,3]. However, the complexity of OSA patients undergoing orthognathic surgery necessitates meticulous preoperative planning that integrates three-dimensional airway assessment, virtual surgical planning, and interdisciplinary collaboration to optimize both functional and aesthetic outcomes [2,4,5].

Despite these advances, accurate modeling of soft tissue behavior and prediction of lateral pharyngeal airway changes remain challenging. Moreover, there is currently no widely accepted standardized or consistent surgical planning protocol defining the optimal magnitude of MMA or the degree of counterclockwise rotation required to achieve predictable, durable airway enlargement and sustained clinical improvement in OSA [6,7,8]. These limitations underscore the need for refinement and standardization of planning algorithms in sleep-related orthognathic surgery [4].

Orthognathic planning for OSA relies primarily on skeletal, dental and soft tissue landmarks and cranial base reference lines to define sagittal jaw relationships. However, these approaches are highly sensitive to head orientation, contributing to variability in surgical planning and outcome prediction [1,2,3,4,5]. In particular, the Frankfort horizontal plane, which is commonly used for orientation of three-dimensional datasets [9], does not consistently correspond to a true horizontal reference in orthognathic patients. These constraints have led to discrepancies with natural head orientation and have prompted growing support for natural head position as a more appropriate reference for contemporary virtual surgical planning.

Importantly, no surgical reference independent of traditional cephalometric landmarks has yet been widely adopted to standardize sagittal planning in OSA-focused orthognathic surgery. In this context, Hernández-Alfaro and colleagues introduced a reproducible anatomical reference associated with favorable functional and aesthetic clinical outcomes, known as the Barcelona line (BL) [10]. The BL is a vertical soft tissue reference derived from the upper incisor–soft tissue plane and passing through the soft tissue nasion in natural head orientation. The BL provides a reproducible framework for individualized anteroposterior positioning of the maxillomandibular complex, independent of intracranial reference planes or population-based cephalometric norms. Its integration into virtual surgical planning protocols has been proposed to support balanced facial outcomes and may be relevant in functional planning for patients with OSA [11].

Therefore, the aim of this retrospective cohort study was to describe the clinical, polysomnographic, skeletal, and three-dimensional airway outcomes of selected adults with moderate-to-severe obstructive sleep apnea (OSA) and dentofacial deformity treated with maxillomandibular advancement (MMA) planned according to the Barcelona line (BL) reference. While previous BL research has focused primarily on orthognathic planning and skeletal and aesthetic outcomes, its application to MMA in patients with OSA and associated functional outcomes remains less well characterized. This study was designed as a feasibility cohort and was not intended to compare the BL with alternative planning methods. The primary outcome was change in apnea–hypopnea index (AHI). Secondary outcomes included changes in Epworth Sleepiness Scale (ESS) scores and three-dimensional upper-airway parameters.

## 2. Materials and Methods

### 2.1. Study Design and Ethical Approval

This was a retrospective cohort study of adult patients with OSA treated with bimaxillary orthognathic surgery planned according to the BL protocol at the Maxillofacial Institute, Teknon Medical Center (Barcelona, Spain). Clinical and radiologic data were retrieved from the institutional database of consecutive cases operated between January of 2018 and December of 2020. The study followed the institutional standards for reporting patient-oriented research. Ethical approval was obtained from the Teknon Medical Center Institutional Review Board (OSAS-OS-2017-CMF-TEK), and all procedures were conducted in accordance with the Declaration of Helsinki (Helsinki, 2024). At the time of surgery, all patients had signed informed consent for the surgical procedures and for the use of their anonymized data for research purposes.

### 2.2. Study Population

Eligible participants were identified by cross-referencing the OSAS registry and the orthognathic surgery database. All eligible patients were consecutively included during the study period to reduce potential selection bias associated with discretionary case selection. Inclusion criteria were age ≥ 18 years; diagnosis of moderate-to-severe OSA based on attended overnight polysomnography, defined as AHI ≥15 events/h according to AASM criteria; presence of dentofacial deformity requiring orthognathic correction; treatment with MMA planned according to the BL reference; and availability of complete preoperative and postoperative CBCT and PSG records at ≥12 months. Exclusion criteria were syndromic or congenital craniofacial anomalies, previous orthognathic or pharyngeal surgery, American Society of Anaesthesiologists (ASA) physical status ≥IV, systemic conditions or therapies capable of compromising bone healing, incomplete imaging or sleep study data, and failure to discontinue continuous positive airway pressure (CPAP) or mandibular advancement device therapy sufficiently before preoperative PSG. This yielded a final sample of 26 patients with OSA operated at the Maxillofacial Institute, Teknon Medical Center, with a uniform BL-based MMA protocol. Demographic data (age, sex, and body mass index), dental classification (Angle class), and OSA severity at baseline were recorded for all subjects.

### 2.3. Polysomnographic Assessment and Outcome Definitions

OSA was confirmed by overnight PSG and AHI, in which OSA and hypopnea events were scored according to the AASM manual, using the recommended criteria for respiratory scoring [12]. Apnea was defined as a ≥90% drop in airflow for ≥10 s; hypopnea was defined as a ≥30% reduction in airflow lasting ≥10 s and associated with ≥3% oxygen desaturation or an arousal. Daytime sleepiness was assessed using the Epworth Sleepiness Scale scores (ESS), a validated 8-item questionnaire with scores from 0 to 24 [13,14]. Preoperative PSG and ESS were performed within a standardized pre-surgical workup period, and postoperative recordings were obtained after completion of the orthognathic treatment and stabilization, with a minimum follow-up of 12 months. Therapeutic response was classified using commonly adopted OSA surgical criteria: treatment success was defined as a ≥50% reduction in AHI compared with baseline combined with a postoperative AHI < 20 events/h and cure was defined as a postoperative AHI < 5 events/h.

### 2.4. Orthognathic Workflow and Preoperative Protocol

All patients were managed according to a standardized orthognathic workflow. The preoperative imaging protocol included a CBCT scan (i-CAT, Imaging Sciences International, Hatfield, PA, USA) obtained with the patient seated in natural head position (NHP). A thin interocclusal wax record was used to avoid tooth-to-tooth contact when necessary. Also, CBCT was repeated at an early postoperative time and at 12 months. For the OSA analysis, the preoperative scan (T0) and the long-term scan T1, ≥12 months postoperatively were used. All DICOM datasets were imported into Dolphin Imaging (version 11.x, Dolphin Imaging & Management Solutions, Chatsworth, CA, USA) for 3D analysis.

### 2.5. Virtual Surgical Planning

Virtual surgical planning incorporated a three-dimensional cephalometric analysis of the maxillomandibular sagittal relationship with the patient oriented in NHP. CBCT datasets were reoriented to NHP using standardized photographic registration to the true horizontal and vertical planes. Following reorientation, sagittal skeletal relationships were quantified using linear measurements rather than intracranial angular references. Specifically, the true vertical plane was established, and the linear distance between Points A and B was measured relative to this vertical reference [15].

The same vertical reference was subsequently applied as the BL and used to guide individualized anteroposterior positioning of the maxillomandibular complex. The relationship of the upper incisor and soft-tissue pogonion to the BL was used to determine the required advancement. The upper incisor was planned slightly anterior to the BL (typically 1–4 mm), while mandibular positioning was adjusted to balance facial aesthetics, occlusal relationships, and airway objectives. Linear and angular skeletal parameters (SNA, SNB, ANB, pogonion advancement, and occlusal plane orientation) were obtained from the three-dimensional cephalometric module. Intermediate and final occlusal splints were fabricated in-house using a CAD/CAM workflow. The planned advancement at pogonion (Pg) was recorded in millimeters for subsequent analysis (Figure 1).

### 2.6. Surgical Technique

All procedures were performed under general anesthesia by the same senior surgeon (FHA) and surgical team. Mandibular surgery consisted of a bilateral sagittal split osteotomy using an Obwegeser-type technique. Fixation was achieved with a hybrid protocol combining a buccal miniplate with monocortical screws and a retromolar bicortical screw on each side, providing rigid stabilization with controlled rotation of the mandibular segment. The maxillary surgery was performed via a Le Fort I osteotomy, segmented or non-segmented depending on occlusal and transverse requirements. A minimally invasive “twist” approach was used to preserve nasal and midfacial musculoligamentous attachments, consistent with previously published methods. Following repositioning according to the Barcelona line (BL)-based virtual plan, the maxilla was fixed with titanium miniplates and screws. A modified alar cinch suture and V-Y mucosal closure were systematically used to maintain nasal base width and upper lip projection. No concomitant genioglossus advancement procedures were performed.

Intraoperatively, maxillary and mandibular movements were verified clinically and by comparing the surgical field to the BL-based virtual plan. Immediate postoperative management included 24 h inpatient monitoring, continuous cold mask therapy at 17 °C, prophylactic antibiotics and non-steroidal anti-inflammatory drugs, and light guiding elastics for one month of functional rehabilitation. A soft diet was maintained for the same period.

### 2.7. Upper Airway Segmentation and 3D Airway Analysis

For airway analysis, the upper airway was segmented on the 3D CBCT. Airway segmentation was performed using Dolphin software. Total upper-airway volume was recorded preoperatively and postoperatively, and the change in total upper-airway volume was calculated. All measurements were performed by a single calibrated examiner; in cases of uncertainty, segmentation was reviewed by a second experienced observer.

### 2.8. Outcome Variables and Statistical Analysis

The primary outcome variables were the changes from T0 to T1 in AHI and upper airway morphology, together with the proportion of patients achieving surgical success and cure. Secondary outcomes included changes in ESS score, as well as three-dimensional airway changes.

Descriptive statistics were used to summarize the study population. Continuous variables are reported as mean ± standard deviation and categorical variables as absolute numbers and percentages. Preoperative and postoperative comparisons were performed using paired statistical tests. Normality of continuous variables and paired differences was assessed by visual inspection of data distribution. Paired *t*-tests were applied for normally distributed variables. Changes in AHI, ESS scores, and upper-airway volume were analyzed accordingly. Treatment success and cure rates were calculated as proportions with corresponding 95% confidence intervals. The association between pogonion advancement and total upper-airway volume gain was assessed using Pearson correlation. A simple linear regression model was also fitted with airway-volume gain as the dependent variable and pogonion advancement, measured in millimeters, as the independent variable. The coefficient of determination, regression slope, intercept, and *p*-value for the slope were reported. A two-sided *p*-value < 0.05 was considered statistically significant. Given the retrospective feasibility design, no a priori sample-size calculation was performed; the sample comprised all consecutive eligible patients meeting the predefined inclusion criteria during the study period.

## 3. Results

### 3.1. Study Population

Twenty-six consecutive patients with OSA underwent MMA planned with the BL reference. The mean age was 37.2 ± 6.0 years, and 17 patients (65.4%) were male. Mean body mass index was 28.8 ± 3.5 kg/m^2^ (Table 1).

### 3.2. Surgical Movements and Skeletal Changes

The mean advancement at the pogonion was 19.0 ± 4.7 mm, with a range from 9 to 28 mm (Figure 2 and Figure 3) (Table 2).

### 3.3. Sleep-Related Outcomes

Mean AHI decreased significantly from 45.8 ± 11.6 events/h preoperatively to 6.6 ± 6.6 events/h postoperatively, corresponding to a mean absolute reduction of 39.1 events/h (95% CI 34.6–43.7; *p* < 0.001) and a mean relative reduction of 85.6 ± 10.9%. Treatment success (defined as a postoperative AHI <20 events/h with a reduction ≥50% from baseline) was achieved in 25 of 26 patients (96.2%; 95% CI 81.1–99.3), and cure (defined as a postoperative AHI <5 events/h) was observed in 22 of 26 patients (84.6%; 95% CI 66.5–93.9).

Subjective daytime sleepiness improved markedly following surgery. ESS scores decreased from 12.2 ± 1.2 preoperatively to 1.5 ± 0.4 postoperatively, with a mean reduction of 10.7 points (95% CI 10.2–11.2; *p* < 0.001) (Figure 2 and Table 2). No minor or major complications noted.

### 3.4. Upper Airway Morphology

Total upper airway volume increased from 26.46 ± 2.88 mm^3^ to 45.32 ± 5.41 mm^3^, corresponding to a mean gain of 18.86 ± 5.10 mm^3^ (95% CI 16.80–20.92; *p* < 0.001). Pogonion advancement was strongly correlated with airway-volume gain (r = 0.80; *p* < 0.001). Pogonion advancement was strongly and positively correlated with total upper-airway volume gain (Pearson r = 0.80, *p* < 0.001). On simple linear regression, pogonion advancement explained 64.0% of the variability in airway-volume gain (R^2^ = 0.64). Each additional millimeter of pogonion advancement was associated with an estimated 0.86 mm^3^ increase in total upper-airway volume gain, according to the regression equation: airway-volume gain = 2.52 + 0.86 × pogonion advancement.

## 4. Discussion

The OSA disorder significantly impairs quality of life and is strongly associated with systemic comorbidities [16,17,18]. Although CPAP remains the first-line therapy, long-term effectiveness is limited by poor adherence, with reported noncompliance rates exceeding 40–50% in real-world settings [19,20]. Consequently, surgical interventions have emerged as effective alternative treatment for moderate-to-severe OSA, demonstrating substantial reductions in AHI and durable long-term outcomes [21,22,23].

The aim of surgical treatment of OSA consists of correcting anatomical contributors to upper airway. Yet, contemporary evidence supports a site-specific and phenotype-driven surgical strategy, reflecting the heterogeneous and frequently multilevel nature of airway obstruction in OSA [24,25]. Nasal procedures, including septoplasty and turbinate reduction, primarily improve airflow and adherence to positive airway pressure but rarely achieve meaningful reductions in AHI when performed alone. Similarly, soft tissue procedures such as uvulopalatopharyngoplasty, tonsillectomy, tongue base reduction, and hyoid suspension target retropalatal and hypopharyngeal collapse. However, their outcomes mostly remain variable and highly dependent on patient selection, with generally lower and less durable success rates compared with skeletal interventions [24,25].

Among skeletal surgical interventions, MMA produces advances in both jaws, often leading to global and simultaneous enlargement of the retropalatal and retroglossal airway. This procedure displaces the tongue anteriorly as well as the soft palate and associated pharyngeal structures, thereby addressing multilevel obstruction within a single procedure [25,26]. Hence, this skeletal modification directly alters the anatomical boundaries of the airway, explaining the superior and durable reductions in AHI reported in the literature, often exceeding 80% in appropriately selected patients [26].

Recent studies suggest that surgical therapies remain underutilized in routine practice, largely due to treatment preferences and comorbidity profiles rather than limited efficacy, highlighting the need for improved identification of candidates for definitive anatomical correction [27]. In this context, MMA occupies a unique role as an optimal surgical intervention with consistent curative potential, supporting its consideration as a definitive treatment option in appropriately selected patients. However, the literature lacks a standardized, reproducible planning protocol based on stable craniofacial landmarks for MMA in patients with OSA. This challenge is further fueled by known limitations in three-dimensional CBCT landmark identification and airway segmentation, which are influenced by head posture, patient motion, and software-dependent thresholding parameters.

Consequently, the present study reports outcomes of MMA performed using a BL-guided planning approach, consistent with a soft tissue relayed sagittal reference previously described in orthognathic surgery. The baseline for the BL was first introduced by Hernández-Alfaro F. related to the upper incisor to soft-tissue plane (UI-STP), a soft-tissue-based reference that quantifies facial projection. Prior studies have demonstrated that the UI-STP and its evolution into the BL provide a simple, reproducible alternative to conventional intracranial cephalometric analyses, with strong correlation to facial harmony and aesthetic preference [28].

Thus, using this protocol, the present series demonstrated significant treatment and cure rates that are within the upper range of those reported in the literature for MMA in appropriately selected patients. Indeed, in a large meta-analysis, Zaghi et al. reported a surgical success rate of 85.5% and a cure rate of 38.5% following MMA for OSA. Similarly, Zhou et al. reported success and cure rates of 85.0% and 46.3%, respectively, when comparing MMA with multilevel soft tissue surgery [3,29].

Moreover, in the present cohort, MMA planned with the BL reference was associated with a marked reduction in mean AHI accompanied by a clinically meaningful improvement in daytime sleepiness. A significant increase in total upper-airway volume was also observed following MMA. However, one patient did not meet the predefined treatment success criterion. This patient had the highest baseline AHI in the cohort, the largest pogonion advancement, and a postoperative AHI that remained in the moderate range despite a relative reduction. This case suggests that skeletal advancement magnitude alone may not fully explain postoperative response and that non-anatomical contributors, such as sleep phenotype, residual multilevel collapse, ventilatory instability, etc., may influence outcomes.

Importantly, given the study design, no conclusions can be drawn regarding the independent contribution of the planning reference versus advancement magnitude or patient selection. Additionally, despite the well-documented efficacy of MMA for functional and aesthetic correction, variability in sagittal planning remains a major limitation in orthognathic and sleep surgery literature. Traditional cephalometric analyses rely on intracranial reference planes or population norms that are often poorly reproducible in three dimensions and highly sensitive to head orientation, limiting inter-study comparability and predictive accuracy. In response to this limitation, the BL has been proposed and validated as a soft-tissue-based, vertical reference anchored to the soft tissue nasion in natural head position, independent of skeletal morphology or occlusal class. Multiple studies have demonstrated that positioning the maxillomandibular complex relative to the BL yields consistent, reproducible sagittal outcomes with high surgical accuracy and strong correlation with aesthetics and soft tissue harmony [30,31].

Longitudinal three-dimensional analyses have shown that BL-guided planning results in predictable mandibular positioning and stability after bimaxillary surgery, with the mandible more frequently advanced rather than set back, thereby avoiding posterior airway compromise [32]. Likewise, BL-based repositioning produces improved upper lip projection, vermilion eversion, and reduction of nasolabial folds, supporting its role as a functional as well as aesthetic reference [33]. Collectively, prior studies suggest that the BL can be applied as a reproducible soft-tissue-based reference in orthognathic planning and its use may facilitate consistent sagittal decision-making that may lead to reproducible planning reference in MMA for OSA.

However, the present study was not designed to determine whether BL-guided planning confers greater long-term skeletal stability than conventional planning methods. Such an effect cannot be inferred from the current findings and should be evaluated in prospective comparative studies using standardized longitudinal three-dimensional skeletal measurements. Also, the BL is a soft-tissue-based reference and may be influenced by individual anatomical characteristics, including upper-incisor inclination, sex-related facial differences, and population-specific craniofacial morphology [34]. Furthermore, the relatively young age and lower BMI of this selected cohort may limit extrapolation to older or more obese populations with OSA. These factors should be considered when applying the BL and warrant validation in larger, more diverse populations. As such, this is a retrospective design and the relatively small sample size from a single tertiary referral center may limit the generalizability of the findings.

Because all procedures were performed at a single center by the same experienced surgical team, the homogeneous cohort and standardized BL-guided workflow minimized procedural variability but also limit external validity. These results may not be reproducible at centers with different surgical expertise, workflows, planning protocols, or patient populations. Thus, multicenter studies with multiple surgeons are therefore needed to assess the reproducibility and generalizability of BL-guided planning.

Also, the absence of a control group planned with alternative sagittal references precludes any comparison of the BL with conventional strategies. Additionally, polysomnographic follow-up, although performed at a minimum of 12 months, does not capture very long-term outcomes or the potential influence of aging or weight changes on OSA recurrence, but this is a common reported outcome in similar studies. Similarly, the independent influence of orthodontic treatment was not specifically assessed. Postoperative BMI and weight changes were not recorded as study variables; therefore, their potential influence on postoperative OSA outcomes could not be evaluated.

Moreover, formal intra- and inter-observer reliability of the CBCT airway measurements was not assessed, which represents an additional limitation. A dedicated longitudinal analysis of facial soft-tissue changes was also not performed, limiting the assessment of detailed aesthetic outcomes of MMA, including upper-lip and midfacial soft-tissue changes, which should be further investigated.

Furthermore, the relatively large magnitude of skeletal advancement represents a potential confounding factor, as the observed airway and polysomnographic improvements may partly reflect the effect of advancement magnitude itself rather than an independent effect of BL-guided planning. The present study design does not allow these effects to be disentangled.

In addition, future work should compare BL-guided planning directly against alternative sagittal planning approaches. Thus, prospective, multicenter comparative studies spanning different surgeons and institutions would help clarify whether the BL independently enhances planning reproducibility, surgical accuracy, or clinical outcomes beyond what advancement magnitude, patient selection, and surgical expertise already explain. Where practical, randomized controlled designs could offer a more rigorous test of the BL’s independent contribution.

## 5. Conclusions

In this retrospective single-center cohort of selected adults with moderate-to-severe OSA and dentofacial deformity, MMA planned using the BL reference was associated with substantial improvements in AHI, ESS, and three-dimensional airway parameters. However, these findings do not establish that the BL protocol is superior or equivalent to conventional planning methods, nor do they isolate the independent effect of the BL reference from advancement magnitude, patient selection, or surgical expertise. The results support the feasibility of BL-guided MMA within a standardized institutional workflow and justify prospective comparative multicenter studies.

## Figures and Tables

**Figure 1:**
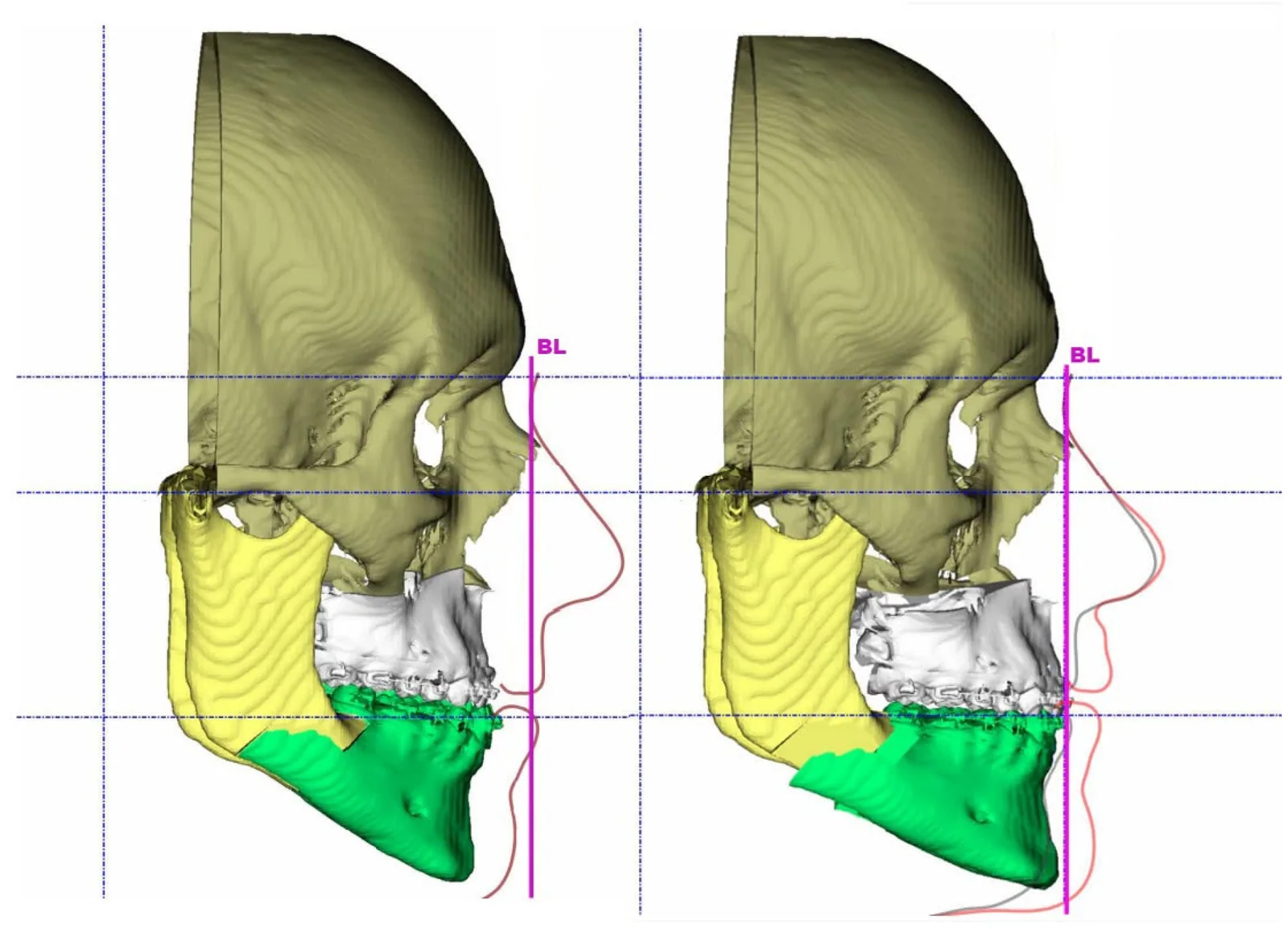
BL-guided virtual surgical plan: natural head orientation superimposition for Barcelona line (BL) demarcation. Three-dimensional virtual surgical planning oriented in natural head position showing demarcation of the Barcelona line (BL). The BL is defined as a vertical soft tissue reference passing through the soft tissue nasion and derived from the upper incisor–soft tissue plane. The relationship between the maxillomandibular complex and the BL was used to guide individualized anteroposterior positioning during virtual planning of maxillomandibular advancement.

**Figure 2:**
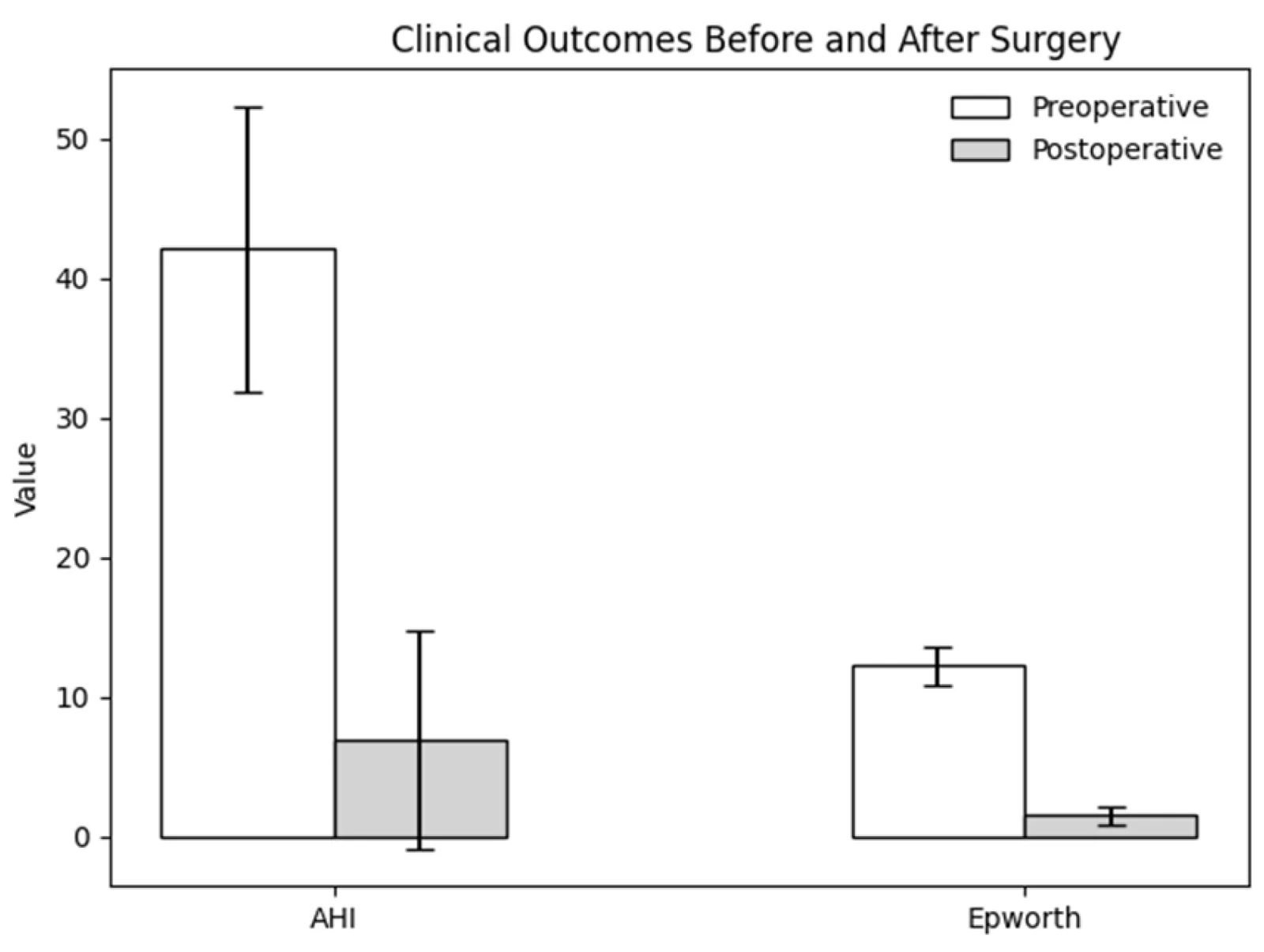
Clinical outcomes pre- and post-BL-guided maxillomandibular advancement. Bar graphs illustrating preoperative and postoperative apnea–hypopnea index (AHI) and Epworth Sleepiness Scale scores. Bars represent mean values and error bars indicate standard deviation, showing marked postoperative improvements in objective sleep parameters and daytime sleepiness.

**Figure 3:**
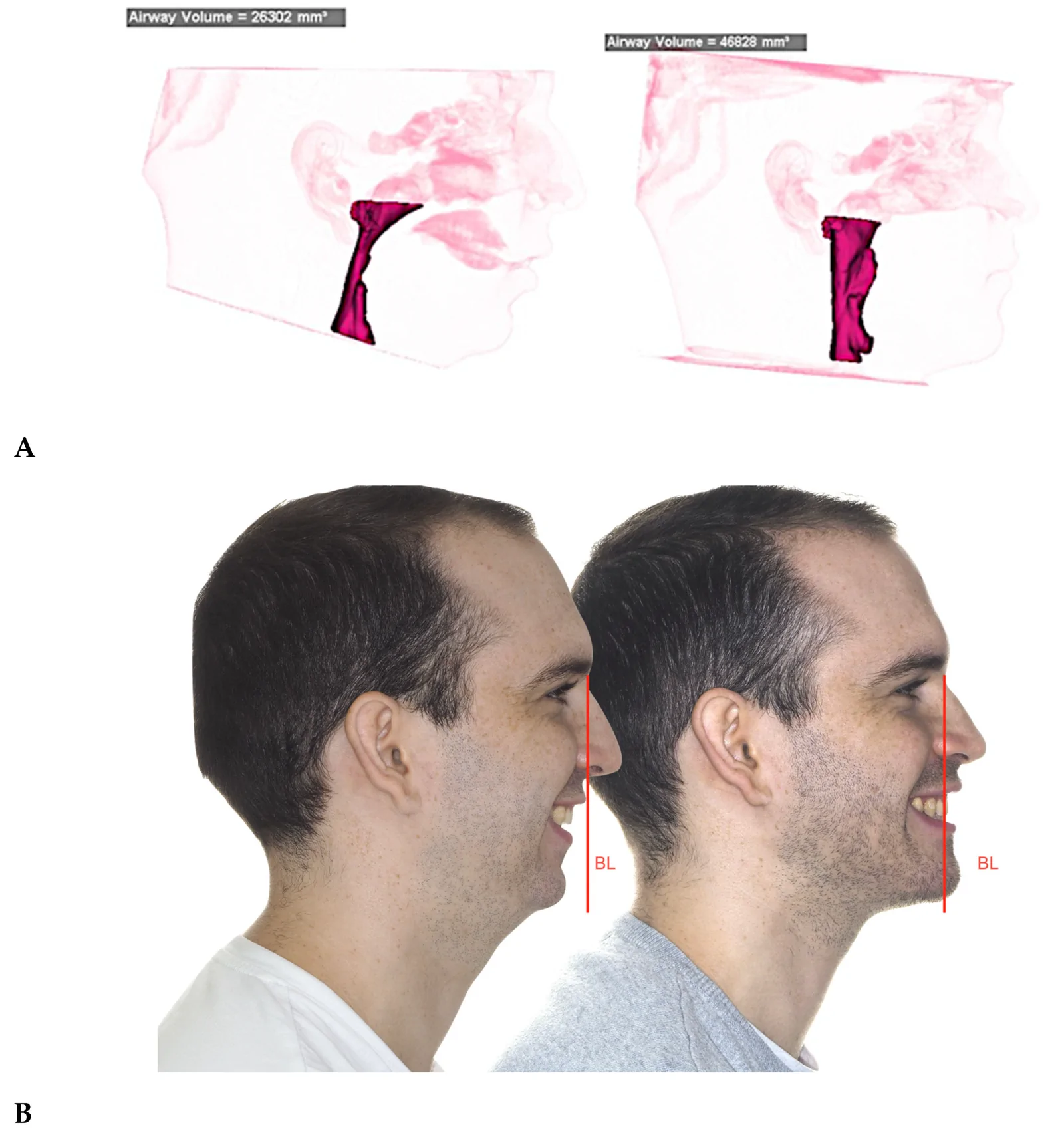
Pre- and post-BL-guided maxillomandibular advancement. Skeletal changes following BL-guided maxillomandibular advancement. (**A**) Three-dimensional CBCT reconstructions of the upper airway before (**left**) and after (**right**) Barcelona line-guided maxillomandibular advancement. The segmented upper airway is highlighted, illustrating postoperative enlargement of the pharyngeal airway. Total airway volume (mm^3^) is displayed for each time point, demonstrating an increase following surgery. (**B**) Preoperative and postoperative lateral views illustrating skeletal repositioning achieved with Barcelona line-guided maxillomandibular advancement. Advancement of the mandible and maxilla relative to the BL resulted in anterior displacement of the maxillomandibular complex.

**Table 1:** Baseline demographic and clinical characteristics of the study population.

Case Nº	Age (Years Old)	Gender	BMI
1	46.2	Male	31
2	31.9	Male	26.8
3	42.9	Male	23.7
4	33.6	Female	23.4
5	37.2	Male	30.4
6	40.5	Male	36.4
7	32.2	Male	30.1
8	34.6	Male	27.5
9	41.8	Female	35.3
10	33.7	Male	30
11	43.6	Male	29.5
12	45.3	Male	30
13	47.6	Male	28.8
14	28.6	Male	28.2
15	36.1	Male	24.6
16	37.5	Female	30.8
17	36.4	Female	28.9
18	45.2	Female	33
19	35.6	Female	28
20	35.2	Female	23.1
21	28.9	Female	28.9
22	34.4	Male	34.6
23	29.6	Male	28
24	46.5	Male	26.4
25	34.1	Female	25.4
26	28	Male	25.8
Overall cohort (n = 26)	37.2 ± 6.0	Male = 17 (65.4%)	28.8 ± 3.5

Values are presented as mean ± standard deviation unless otherwise indicated. Percentages are based on the total sample size (n = 26). BMI, body mass index.

**Table 2:** Skeletal changes and individual sleep-related outcomes following Barcelona line-guided maxillomandibular advancement.

Case Nº	Pogonion Advancement (mm)	Pre Airway Volume mm^3^	Post Airway Volume mm^3^	Airway Volume Gain (mm^3^)	Pre OP ESS	Post OP ESS	Pre OP AHI	Post OP AHI	Reduction (%) AHI	Treatment Success	OSA Cure
1	9	29.013	42.823	13.81	12.1	1.5	42.4	15	64.6	Yes	No
2	28	26.252	54.599	28.347	12.2	1.3	45.1	4.1	90.9	Yes	Yes
3	13.6	25.583	36.949	11.366	10.3	1.1	24	4.8	80	Yes	Yes
4	22.5	28.905	54.464	25.559	10.7	1.1	46.7	4.8	89.7	Yes	Yes
5	12.5	23.805	32.717	8.912	11	1	42.3	12.4	70.7	Yes	No
6	20.6	26.837	42.04	15.203	12.2	1.6	60.2	4.1	93.2	Yes	Yes
7	16.6	31.052	46.579	15.527	11.8	1.5	44.8	4.3	90.4	Yes	Yes
8	22.8	27.278	52.396	25.118	14.9	0.9	52.2	4.2	92	Yes	Yes
9	17.4	27.888	45.779	17.891	13.8	1.9	42.6	4.8	88.7	Yes	Yes
10	23.8	17.735	41.175	23.44	13.4	2.3	24	3.6	85	Yes	Yes
11	17.4	26.228	48.469	22.241	11.5	0.8	45.3	4.8	89.4	Yes	Yes
12	24.2	26.02	51.917	25.897	12.7	1.7	44.9	4	91.1	Yes	Yes
13	18.4	21.846	41.908	20.062	9.8	2.2	36.1	4.2	88.4	Yes	Yes
14	25.1	26.191	50.971	24.78	14.4	1	50.4	3.7	92.7	Yes	Yes
15	18.9	28.356	46.99	18.634	11.4	1.1	43.1	3.7	91.4	Yes	Yes
16	14.2	28.524	44.762	16.238	12.7	1.5	63.5	4.3	93.2	Yes	Yes
17	19.1	28.489	44.766	16.277	13.2	1.5	37.2	4.6	87.6	Yes	Yes
18	12.5	28.047	37.917	9.87	12.4	1.3	50.5	14.8	70.7	Yes	No
19	24.4	30.415	52.41	21.995	11.4	1.9	48.2	3.3	93.2	Yes	Yes
20	20.3	22.499	40.783	18.284	11.6	1.7	42.8	4.1	90.4	Yes	Yes
21	13.3	28.129	41.654	13.525	12.6	1.2	66	35	47	No	No
22	24.4	26.998	43.827	16.829	11.4	1.7	38.3	4.8	87.5	Yes	Yes
23	21.7	27.209	45.716	18.507	12.2	1.8	29.8	4.4	85.2	Yes	Yes
24	16.4	26.107	47.392	21.285	11.3	1.4	73.3	4.8	93.5	Yes	Yes
25	16.3	26.075	45.217	19.142	13.8	1.9	54	4.4	91.9	Yes	Yes
26	20.6	22.452	44.177	21.725	12.4	2.1	41.9	4.8	88.5	Yes	Yes
Overall cohort (n = 26)	19.0 ± 4.7	26.459 ± 2.883	45.32 ± 5.41	18.864 ± 5.099, CI 16.80 to 20.92	12.2 ± 1.2	1.5 ± 0.4	45.8 ± 11.6 events/h	6.6 ± 6.6 events/h	85.6 ± 10.9%	25/26, 96.2%, CI 81.1–99.3	22/26, 84.6%, CI 66.5–93.9

Values are presented as mean ± standard deviation unless otherwise indicated. Percentages are based on the total sample size (n = 26). BL, Barcelona line; ESS, Epworth Sleepiness Scale; AHI, apnea–hypopnea index. Treatment success was defined as a ≥50% reduction in AHI combined with a postoperative AHI < 20 events/h; cure was defined as a postoperative AHI < 5 events/h.

## Data Availability

The data that support the findings of this study are available from the corresponding author upon reasonable request.

## Abbreviations

The following abbreviations are used in this manuscript:

OSA	Obstructive sleep apnea
MMA	Maxillomandibular advancement
BL	Barcelona Line
AHI	Apnea–hypopnea index
ESS	Epworth Sleepiness Scale
CBCT	Cone-beam computed tomography
PSG	Polysomnography
CPAP	Continuous positive airway pressure
AASM	American Academy of Sleep Medicine
ASA	American Society of Anesthesiologists
NHP	Natural head position
DICOM	Digital Imaging and Communications in Medicine
CAD/CAM	Computer-aided design/computer-aided manufacturing
Pg	Pogonion
SNA	Sella–Nasion–A point angle
SNB	Sella–Nasion–B point angle
ANB	A point–Nasion–B point angle
T0	Preoperative time point
T1	Postoperative follow-up time point
BMI	Body mass index
CI	Confidence interval
UI-STP	Upper incisor to soft-tissue plane
IRB	Institutional Review Board
CC BY	Creative Commons Attribution license
3D	Three-dimensional
V-Y	V-Y mucosal closure
